# Impact of long-COVID on health-related quality of life in Japanese COVID-19 patients

**DOI:** 10.1186/s12955-022-02033-6

**Published:** 2022-08-19

**Authors:** Shinya Tsuzuki, Yusuke Miyazato, Mari Terada, Shinichiro Morioka, Norio Ohmagari, Philippe Beutels

**Affiliations:** 1 AMR Clinical Reference Center, National Center for Global Health and Medicine, 1-21-1 Toyama, Shinjuku-ku, Tokyo, 162-8655 Japan; 2 Centre for Health Economics Research & Modelling Infectious Diseases, Vaccine & Infectious Disease Institute, Faculty of Medicine and Health Sciences, University of Antwerp, Prinsstraat 13, 2000 Antwerp, Belgium; 3 Disease Control and Prevention Center, National Center for Global Health and Medicine, Tokyo, Japan; 4 Center for Clinical Sciences, National Center for Global Health and Medicine, Tokyo, Japan; 5 Emerging and Re-Emerging Infectious Diseases, Graduate School of Medicine, Tohoku University, Sendai, Japan; 6 School of Public Health, University of New South Wales, Sydney, Australia

**Keywords:** COVID-19, Disease burden, Quality of life, Long-COVID

## Abstract

**Background:**

The empirical basis for a quantitative assessment of the disease burden imposed by long-COVID is currently scant. We aimed to inform the disease burden caused by long-COVID in Japan.

**Methods:**

We conducted a cross sectional self-report questionnaire survey. The questionnaire was mailed to 526 eligible patients, who were recovered from acute COVID-19 in April 2021. Answers were classified into two groups; participants who have no symptom and those who have any ongoing prolonged symptoms that lasted longer than four weeks at the time of the survey. We estimated the average treatment effect (ATE) of ongoing prolonged symptoms on EQ-VAS and EQ-5D-3L questionnaire using inverse probability weighting. In addition to symptom prolongation, we investigated whether other factors (including demography, lifestyle, and acute severity) were associated with low EQ-VAS and EQ-5D-3L values, by multivariable linear regression.

**Results:**

349 participants reported no symptoms and 108 reported any symptoms at the time of the survey. The participants who reported any symptoms showed a lower average value on the EQ-VAS (69.9 vs 82.8, respectively) and on the EQ-5D-3L (0.85 vs 0.96, respectively) than those reporting no symptoms considering the ATE of ongoing prolonged symptoms. The ATE of ongoing prolonged symptoms on EQ-VAS was − 12.9 [95% CI − 15.9 to − 9.8], and on the EQ-5D-3L it was − 0.11 [95% CI − 0.13 to − 0.09], implying prolonged symptoms have a negative impact on patients’ EQ-VAS and EQ-5D-3L score. In multivariable linear regression, only having prolonged symptoms was associated with lower scores (− 11.7 [95% CI − 15.0 to − 8.5] for EQ-VAS and − 0.10 [95% CI − 0.13 to − 0.08] for EQ-5D-3L).

**Conclusions:**

Due to their long duration, long-COVID symptoms represent a substantial disease burden expressed in impact on health-related quality of life.

## Background

Coronavirus disease 2019 (COVID-19) caused by the SARS-CoV-2 virus, has become a global health threat [1, 2]. Not only its acute phase of disease, but so-called “long-COVID” is also a cause of substantial disease burden [3, 4]. A systematic review reported that 80% of patients developed one or more long-term symptoms and the prevalence of 55 long-term effects of COVID-19 [5].

There is no clear definition of long-COVID so far, however, the National Institute for Health and Care Excellence (NICE) in The UK defined it as “signs and symptoms that develop during or following an infection consistent with covid-19 and which continue for more than four weeks and are not explained by an alternative diagnosis” [6]. This term includes ongoing symptomatic COVID-19, from four to 12 weeks post-infection, and post-COVID-19 syndrome, beyond 12 weeks post-infection [7].

The symptoms of long-COVID are various and often different from the acute phase of COVID-19. Miyazato and colleagues reported that the mean time from COVID-19 symptom onset to the emergence of alopecia was 58.6 days and one of patients presented dysosmia after 92 days after symptom onset [8]. Other symptoms such as general fatigue [9, 10], respiratory symptoms [11, 12], cognitive and mental health disorder [13, 14], and so forth [15, 16] have been reported as long-COVID.

Considering its chronic phase, the disease burden of COVID-19 should be larger than that of other respiratory infections due to length and variety of the symptoms. However, the empirical basis for a quantitative assessment of the disease burden imposed by long-COVID is currently scant.

As already mentioned, COVID-19 is one of the greatest global health crises, of an infectious disease that will eventually become endemic, quantitative evaluations of its disease burden are necessary to appropriately assess the impact of interventions. The burden of Long-COVID-19 should be assessed separately from acute COVID-19 because it has clearly distinct characteristics, as part of the disease burden caused by COVID-19.

Malik and colleagues reported a meta-analysis about post-acute COVID-19 syndrome and the health-related quality of life (HRQoL) [17]. However, their results did not include HRQoL between 0 and 1, as single indicator of health utility. Tran and colleagues investigated the validity of impact tools of long-COVID, and they evaluated the impact of long-COVID quantitatively [18], nevertheless, their main interest is not HRQoL itself but to validate their own tool. Although Tabacof and colleagues also assessed the HRQoL of long-COVID patients [19], they focused on rather each component of EQ-5D and had no control group. Fink and colleagues evaluated the correlation between persistent symptoms of pediatric COVID-19 and HRQoL then the target population was different [20].

As described above, the quantitative evaluation of HRQoL for long-COVID adults as a single indicator of health utility which can easily be applied to more comprehensive study such as cost-effectiveness analysis is still scarce. Our study aims to estimate an important part of the disease burden caused by COVID-19, in order to appreciate the potential impact of interventions against it.

## Methods

### Settings

We conducted a cross-sectional, retrospective survey in which a self-report questionnaire was mailed in April 2021 with two reminders 2 weeks and 1 month later to eligible participants. Potential participants were recruited from the people who visited the outpatient service of the Disease Control and Prevention Center (DCC) in National Center for Global Health and Medicine (NCGM) between 1st February 2020 and 31st March 2021, in order to obtain pre-donation screening test for COVID-19 convalescent plasmapheresis (Another study named “Collection and antibody measurement of Convalescent plasma foreseeing the use for COVID-19 treatment”). i.e., although the questionnaire survey was conducted in April 2020, all the participants have a documented history of COVID-19 at least eight weeks before they visited the outpatient service. The visitors of the outpatient service were voluntarily recruited and 526 participants were included in the study. Visitors who were younger than 20 years old were excluded from the survey. The minimum time from symptom onset or diagnosis of COVID-19 to the questionnaire survey was 56 days. Participants were requested to complete and return the questionnaire and 457 of 526 (86.9%) participants completely answered the questionnaire and were included in the analysis.

### Ethics approval

According to local ethical guidelines, providing responses to the questionnaire was considered as providing participant consent. This study was reviewed and approved by the Ethics Committee of the Center Hospital of the NCGM (NCGM-G-004121-00).

### Measures

EQ-5D-3L and EQ-VAS were used as outcome measures. EQ-5D-3L questionnaire comprises the following five dimensions: mobility, self-care, usual activities, pain/discomfort and anxiety/depression and each dimension has three levels: no problems, some problems, and extreme problems. The subject is asked to answer each question, and the decision results into a score between − 0.6 and 1.0, with 0 corresponding to death, and some exceptional health states having negative values, i.e., being considered by the average person as worse than dead. The subject is also asked to answer EQ-VAS questionnaire, a standard vertical 20 cm visual analogue scale, used in recording an individual’s rating of their overall current health-related quality of life, which scale ranges from 100 ("the best imaginable health state" or "the best health state you can imagine") to 0 ("the worst imaginable health state" or "the worst health you can imagine") was also collected.

We collected information about demographics (age, sex, height, weight, smoking, drinking, pregnancy, and past history of diseases), clinical course of the acute phase of COVID-19 infection (day of onset and/or diagnosis, admission status during the acute phase, use of antivirals/systemic steroids, requirement of supplementary oxygen/mechanical ventilation/extracorporeal membrane oxygenation during admission), and symptoms since onset to the questionnaire survey (fever, fatigue, shortness of breath, joint pain, myalgia, chest pain, cough, abdominal pain, dysgeusia, dysosmia, runny nose, red-eye, headache, sputum, sore throat, diarrhoea, nausea, appetite loss, hair loss, depression, loss of concentration, and memory disturbance). All symptoms were recorded based on self-reporting, with their onset date and duration.

We included age, sex, BMI, smoking, drinking, hypertension, diabetes, chronic obstructive lung diseases, malignancy, use of antivirals, use of systemic steroids, admission status, and severe COVID-19 disease during admission (use of mechanical ventilation or extracorporeal membrane oxygenation during admission), according to the definition by a report of national registry data in Japan [21]) as confounding factors.

### Statistical analysis

The sample size for the linear regression model was calculated by F test [22]. The F test has numerator and denominator degrees of freedom. The numerator degrees of freedom, u, is the number of coefficients (minus the intercept). In our case, $u = 12$ however at the time of calculation, we set $u = 15$. The denominator degrees of freedom, $v$, is the number of error degrees of freedom:

$$v = n - u - 1$$

This implies.

$$n = v + u + 1.$$

The effect size, ${f}^{2}$, is ${R}^{2}/(1-{R}^{2})$, where ${R}^{2}$ is the coefficient of determination, in other words, the “proportion of variance explained”. We used ${f}^{2}=0.15$ which was recommended by Cohen [22] and set the level of significance at 0.05 and power at 0.80. As a result, we obtained $v=122.4$ and the required sample size was $122.4 + 15 + 1 \cong 139$.

Two-sided *p* values of < 0.05 were considered to show statistical significance. All analyses were conducted by R, version 4.0.5 [23].

Answers were classified into two groups; participants who have no ongoing prolonged symptoms and those who have any ongoing prolonged symptoms. “Ongoing prolonged symptom” was defined as symptoms lasted longer than four weeks from the onset of acute phase of COVID-19 infection (i.e., “long-COVID” condition defined in [6]), and, presented at the time of the survey. We evaluated the average treatment effect of ongoing prolonged symptoms on EQ-VAS, which is a measurement instrument that tries to measure the self-reported health status with the range between 0 and 100. and HRQoL values estimated by the EQ-5D-3L questionnaire [24] using the Japanese value set [25].

We used inverse probability weighting (IPW) method with propensity score which was calculated by multivariate logistic regression model predicting the likelihood of having ongoing prolonged symptoms [26, 27]. The standardized mean difference and variance ratio were used to measure covariate balance, and an absolute standardized difference above 10% and variance ratio over 2.0 was interpreted as a meaningful imbalance [28].

Additionally, we investigated factors associated with low EQ-VAS and EQ-5D-3L index values other than ongoing prolonged symptoms by linear regression model. Multicollinearity was examined by variance inflation factor (VIF) and VIF ≥ 2.5 as an indicator of multicollinearity [29].

## Results

The left side of Table 1 shows the basic characteristics of the participants. 457 participants recovered from acute phase of COVID-19 and 108 of them presented at least one ongoing prolonged symptom(s). The proportion of female was larger in “Any symptom” group than that in “No symptom” group. There was no substantial difference between the two groups in terms of their age, medical history, admission status and days from symptom onset/diagnosis to the survey. About a half of participants once admitted to hospitals due to acute phase COVID-19. Crude comparison of EQ-VAS and EQ-5D-3L index showed that “Any symptom” group had lower EQ-VAS and EQ-5D-3L index than the “No symptom” group did (EQ-VAS: 70 vs 85, EQ-5D-3L index: 0.81 vs 1.0, respectively). The right side of Table 1 describes the characteristics of the data after propensity score weighting. 95 of “Any symptom” group and 296 of “No symptom” group were included, and other participants were discarded because of missing items.

**Table 1:** Characteristics of participants before and after propensity score weighting

	All				Propensity score weighted			
	No symptom	Any symptom	SMD	Variance ratio	No symptom	Any symptom	SMD	Variance ratio
Number	349	108			296	95		
Age	47 [48, 39–55]	47 [47, 40–54]	0.001	1.207	46.3 [11.0]	46.0 [10.0]	0.028	1.198
Male	188 (53.9)	38 (35.2)	< 0.383	1.083	147.7 (49.9)	47.4 (49.9)	0.003	1.0
BMI	23.7 [23.2, 21.1–25.6]	23.9 [23.4, 20.9–26.9]	0.163	1.646	23.8 [4.1]	23.6 [4.4]	0.037	1.165
Smoking	130 (37.2)	38 (35.5)	0.036	1.014	103 (34.8)	33.4 (35.2)	0.004	1.0
Drinking	290 (83.1)	86 (79.6)	0.089	1.162	246 (83.1)	79.7 (83.9)	0.008	1.0
Hypertension	52 (14.9)	14 (13.0)	0.056	1.117	39.1 (13.2)	12.5 (13.2)	0.0	1.0
Diabetes	23 (6.6)	5 (4.6)	0.085	1.385	16.6 (5.6)	4.9 (5.2)	0.004	1.0
COPD	3 (0.9)	0 (0.0)	< 0.132	NA	0 (0.0)	0 (0.0)	NA	NA
Malignancy	6 (1.7)	0 (0.0)	0.187	NA	3.8 (1.3)	0 (0.0)	0.013	1.0
Days from symptom onset/diagnosis to the survey*	248.9 [249.0, 148.0–357.0]	250.8 [243.0, 150.0–367.0]	0.018	1.125	NA	NA	NA	NA
Inpatient*	195 (56.0)	63 (58.3)	0.046	1.007	NA	NA	NA	NA
Use of antivirals	62 (18.8)	22 (20.8)	0.049	1.085	49.7 (16.8)	16.7 (17.6)	0.007	1.0
Use of steroids	40 (12.8)	13 (13.3)	0.013	1.037	39.4 (13.3)	12.7 (13.4)	0.001	1.0
Severe disease^†^	7 (2.1)	6 (5.6)	0.183	2.619	7.1 (2.4)	2.2 (2.3)	0.001	1.0
Oxygen support*	44 (12.6)	13 (13.3)	0.027	1.10	NA	NA	NA	NA
EQ-VAS	85 [85, 75–90]	70.4 [70, 60–80]	0.810	1.412	82.8 [13.0]	69.9 [17.3]	0.891	1.773
EQ-5D-3L index score	0.98 [1.0, 1.0–1.0]	0.91 [0.81, 0.77–1.0]	0.845	2.659	0.96 [0.09]	0.85 [0.16]	0.914	3.190

Mean [median, interquartile range/standard deviation] for continuous variables, number (%) for categorical variables

*SMD* standardized mean difference, *BMI* Body Mass Index, *COPD* chronic obstructive pulmonary disease

^*^Not included in calculating propensity score

^†^Use of mechanical ventilation or extracorporeal membrane oxygenation during admission

Table 2 describes the characteristics of prolonged symptoms. We defined “long-COVID” as the status in which any symptoms attributed to SARS-CoV-2 infection last longer than four weeks in our study, regardless of their continued presence at the time the survey was completed. As such, prolonged symptoms in this study indicate “long-COVID” symptoms as defined in [6]. In total 201 of 457 (44.0%) participants reported at least one symptom longer than four weeks after COVID-19 symptom onset. Among these, 73 (16.0%) reported one symptom, 46 (10.1%) two, 47 (10.3%) three, and 35 (7.7%) four or more symptoms. The most common of these prolonged symptoms was general fatigue, which was reported by 58 of 457 (12.7%) participants. The second most common symptom was alopecia, as 55 of 457 (12.0%) participants experienced worse than usual hair loss.

**Table 2:** Details of symptoms lasted longer than four weeks in the participants

	Number	Duration (days)
Fatigue	58 (12.7)	50 [30–60]
Hair loss	55 (12.0)	60 [30–90]
Cough	54 (11.8)	40 [30–60]
Dysosmia	47 (10.3)	45 [30–60]
Dysgeusia	47 (10.3)	35 [30–60]
Shortness of breath	36 (7.9)	42.5 [30–60]
Loss of concentration	34 (7.4)	40 [30–90]
Depression	29 (6.3)	40 [30–60]
Chest pain	18 (3.9)	60 [40–98]
Appetite loss	17 (3.7)	30 [30–60]
Headache	17 (3.7)	44 [30–60]
Memory disturbance	15 (3.3)	60 [30–90]
Sputum	14 (3.1)	43 [30–60]
Fever	11 (2.4)	30 [30–45]
Joint pain	8 (1.8)	48 [30–98]
Myalgia	5 (1.1)	40 [30–60]
Sore throat	5 (1.1)	30 [30–50]
Runny nose	5 (1.1)	30 [30–31]
Red-eye	4 (0.9)	60 [58–75]
Diarrhoea	2 (0.4)	33 [31–34]
Nausea	1 (0.2)	30 [30–30]
Abdominal pain	0 (0.0)	NA

Absolute number (%) for the number of participants, median [interquartile range] for the duration of symptoms

**Fig. 1:**
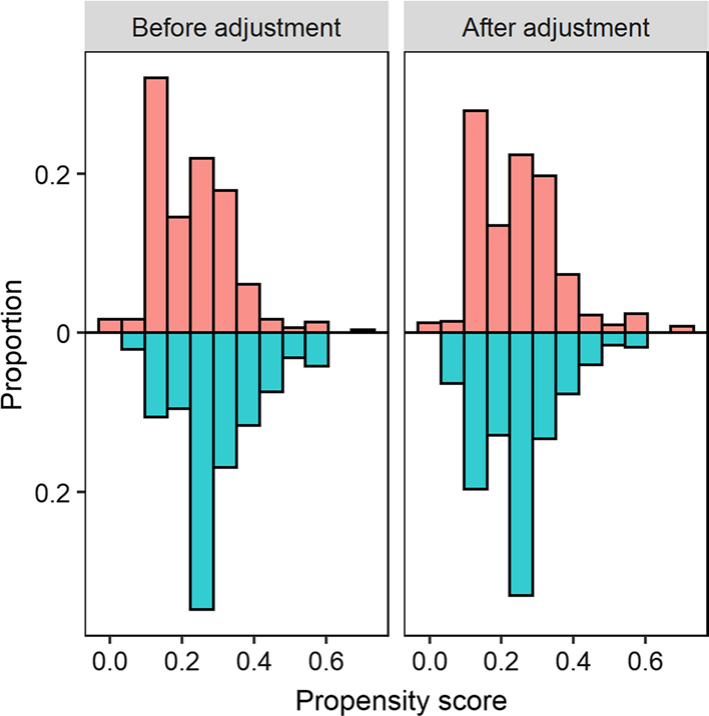
Distribution of propensity score before and after weighting. Red colour represents “No symptom” group and blue colour represents “With symptom” group

**Fig. 2:**
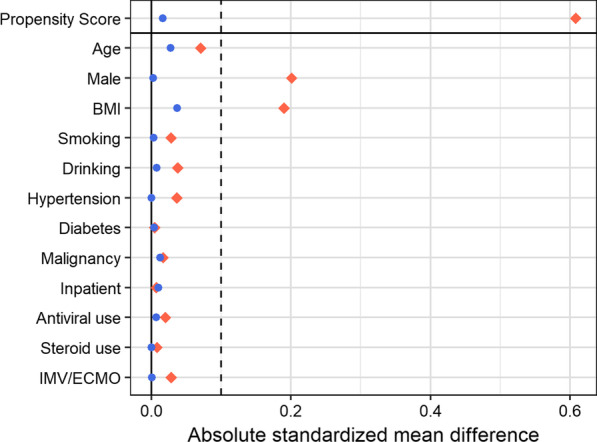
Balance of covariates before and after inverse probability weighting. Red squares represent before adjustment and blue circles represent after adjustment

Figure 1 shows the distribution of propensity scores before and after weighting. Figure 2 shows the balance of covariates before and after weighting. The balance of covariates in both groups improved after IPW weighting. The two groups differed mainly in terms of gender and BMI, which could give rise to confounding factors when comparing their HRQoL measurements. Figure 2 demonstrates that the standardized mean difference in these two factors decreased.

Adjusted EQ-VAS and EQ-5D-3L score comparisons were similar to the unadjusted crude comparisons (Table 3). The ATE of ongoing prolonged symptoms was − 12.9 (95% confidence interval [CI] − 15.9 to − 9.8) on the EQ-VAS, and − 0.11 (95% CI − 0.14 to − 0.09) on the EQ-5D-3L. The differences attributed to the symptoms were larger than the minimally important difference estimated in a previous study (0.048, 95% CI 0.046 to 0.051) [30]. Therefore, prolonged symptoms can be regarded as having clinically significant negative impact on patients’ EQ-VAS and EQ-5D-3L scores.

**Table 3:** Average treatment effect of ongoing prolonged symptoms on EQ-VAS and EQ-5D-3L index

	Intercept	ATE	*P* value
EQ-VAS	82.8 [80.6 to 84.9]	− 12.9 [− 15.9 to − 9.8]	**< 0.001**
EQ-5D-3L index score	0.96 [0.95 to 0.98]	− 0.11 [− 0.13 to − 0.09]	**< 0.001**

*P* values below 0.05 are given in bold

Values are median [95% confidence intervals]

*ATE* average treatment effect

Table 4 shows the results of linear regression analysis about covariates associated with the EQ-VAS (4a) and EQ-5D-3L (4b). Both analyses showed that ongoing prolonged symptoms substantially influence the EQ-VAS and EQ-5D-3L values. Although male sex and steroid use during admission were associated with not lower EQ-VAS scores, no other variable than having ongoing prolonged symptoms was associated with the EQ-5D-3L scores. In both models, all VIF values were below 2.5.

**Table 4:** Results of linear regression analysis about (a) EQ-VAS, (b) EQ-5D-3L index score

Variable	Coefficient	95% confidence interval	*P* value
(a)			
Intercept	85.7	[75.1, 96.4]	**< 0.001**
Ongoing prolonged symptoms	− 11.7	[− 15.0, − 8.5]	**< 0.001**
Age	0.03	[− 0.11, 0.17]	0.642
Male	3.2	[0.2, 6.2]	**0.038**
BMI	− 0.3	[− 0.6, 0.1]	0.125
Smoking	0.2	[− 2.8, 3.2]	0.882
Drinking	2.6	[− 1.1, 6.2]	0.171
Hypertension	− 0.2	[− 4.6, 4.2]	0.924
Diabetes	− 4.7	[− 11.0, 1.6]	0.142
Malignancy	− 4.1	[− 16.2, 8.0]	0.507
Inpatient	− 2.9	[− 5.9, 0.2]	0.066
Use of antivirals	− 1.3	[− 5.7, 3.2]	0.577
Use of steroids	5.3	[0.3, 10.2]	**0.036**
Severe disease^†^	5.4	[− 4.8, 15.6]	0.30
(b)			
Intercept	0.97	[0.89, 1.05]	**< 0.001**
Ongoing prolonged symptoms	− 0.10	[− 0.13, − 0.08]	**< 0.001**
Age	0.00002	[− 0.00001, 0.00003]	0.969
Male	0.02	[− 0.01, 0.05]	0.052
BMI	0.0008	[− 0.001, 0.002]	0.560
Smoking	− 0.01	[− 0.03, 0.01]	0.383
Drinking	0.02	[− 0.01, 0.05]	0.255
Hypertension	− 0.003	[− 0.04, 0.03]	0.860
Diabetes	− 0.05	[− 0.1, 0.01]	0.068
Malignancy	0.002	[− 0.1, 0.1]	0.959
Inpatient	− 0.02	[− 0.04, 0.01]	0.144
Use of antivirals	0.02	[− 0.02, 0.05]	0.301
Use of steroids	0.02	[− 0.02, 0.06]	0.386
Severe disease^†^	0.05	[− 0.03, 0.13]	0.264

*P* values below 0.05 are given in bold

^†^Use of mechanical ventilation or extracorporeal membrane oxygenation during admission

## Discussion

Our results demonstrated that people suffering from the phenomenon we called “long-COVID” showed lower HRQoL. This would be another important aspect of COVID-19 to consider because it implies a heavier disease burden than other influenza like illnesses (ILIs), not only due to its severity but also the characteristics of its chronic phase. In the first place, COVID-19 showed higher case-fatality than other ILIs [31–33]. Additionally, it might cause a substantial burden through accumulated mild disease only.

Furthermore, the frequency and the duration of symptoms due to “long-COVID” are also noteworthy. Our results showed that nearly half of the participants who recovered from acute COVID-19 (201/457) experienced any symptoms lasting more than four weeks. As for participants who required supplementary oxygen support, 32 out of 70 (45.7%) presented any symptoms longer than four weeks. The precise duration of such symptoms was not obvious because more than 100 participants reported that their symptoms were still ongoing. Nevertheless, the symptoms attributed to “long-COVID” often continue for several months. Although the HRQoL valuations for participants who had any “long-COVID” symptoms was better than those previously reported during the acute phase of other ILIs in Japan (0.81 vs 0.66, respectively) [34], the HRQoL losses attributable to “long-COVID” should exceed those due to the acute phase of other ILIs because of its duration.

There are several strengths in this study. First, we evaluated the disease burden of long-COVID using standardised HRQoL instruments yielding HRQoL weights, which can be used as inputs for cost-effectiveness analysis with Quality Adjusted Life Years (QALY) as outcome of interest. This characteristic will be beneficial for further research about COVID-19.

Second, we compared the burden of long-COVID symptoms with the “control” participants who have past histories of the acute phase of COVID-19 infection and no ongoing symptoms due to long-COVID. As described in Background, albeit there are a few studies which investigate the association between HRQoL and long-COVID, most of them did not compare HRQoL of people suffering from long-COVID with healthy controls.

Additionally, our results suggest that prevention is more important in COVID-19 countermeasures than other ILIs because effective treatment of “long-COVID” is not clearly established yet [7, 35]. Although there is no doubt that vaccination against SARS-CoV-2 will reduce the risk of fatal and severe COVID-19 [36–38], its effectiveness against “long-COVID” is not demonstrated yet. This may provide an additional incentive to prevent SARS-CoV-2 infection even in the absence of known risk factors of severe illness.

As our linear regression models demonstrated, there were no definite factors which have negative influence on HRQoL other than ongoing prolonged symptoms. This suggests that lower HRQoL of long-COVID patients can be attributed to these symptoms, and therefore palliative methods against them would be important. With regard to EQ-VAS, male sex and systemic steroid use during admission showed a positive impact on EQ-VAS values. The positive impact of male sex might be attributed to the finding that female COVID-19 patients experience long-COVID more often than male patients [8]. The effect of steroid use during admission is not clear. If treatment during the acute phase of COVID-19 is associated with milder burden than long-COVID, then even mild cases should be treated with appropriate drugs. The impact of treatment during the acute phase of infection on its chronic phase (long-COVID) is an important challenge to address in future research.

In short, symptoms due to long-COVID may be a cause of low HRQoL. Since long-COVID might be an important contributor to future disease burden, effective countermeasures should be considered. At present, there is no established treatment of long-COVID. In anticipation of therapeutic agents for long-COVID, both pharmaceutical (e.g., vaccination) and non-pharmaceutical (e.g., social distancing) preventive interventions remain important.

There are several limitations in our study. First, since our results are based on the questionnaire survey there are some cognitive biases in participants’ responses. The participants answer the questionnaire at least eight weeks after they visited the outpatient service. Given the circumstances, memory recall of the participants might be affected. However, since this study aims to assess the burden of “ongoing” prolonged symptoms, this kind of influence could be trivial.

Second, the potential participants were enrolled from the visitors of outpatient department at the national center hospital of infectious diseases in Japan, implying the study population tend to have had mild disease in their acute period and are comparatively young. Although this can be regarded as a selection bias, long-COVID in relatively young age groups is a serious issue in society, meriting attention in the current social context.

Third, since the participants of this study voluntarily agreed with answering the questionnaire, they can be regarded as having more interest in their own health than that of the general population in Japan. This volunteer bias might be a cause of overestimation in assessment of their prolonged symptoms. In addition, our data about participants’ symptoms were based on self-reported information and not validated by any healthcare professionals. However, we believe that this will not impair the value of our findings substantially because most symptoms attributable to long-COVID are subjective ones such as fatigue, and they are difficult to be validated objectively even if they are assessed by healthcare professionals.

Fourth, there is possible bias caused by non-responders. We do not know why some of participants did not complete the survey. The disease burden of long-COVID could be under/overestimated although the response rate of our survey was quite high (86.9%).

Fifth, we should be careful about the representativeness of the data when we interpret the results because our survey includes a comparatively small number of participants. However, our sample size calculation supported that the number of participants had a sufficient power to detect differences in HRQoL.

As discussed above, there are several sources of bias and we should take care when interpreting the results, nevertheless, also take note that the impact of these limitations can be regarded comparatively small in this study.

Next, we could not take “new variants” into consideration. The difference in severity, infectiousness, and so forth between such new variants and old ones were already reported [39, 40], however, there is no solid evidence about the frequency and the severity of “long-COVID” symptoms in new variants. This should be the subject of future study.

The statistical model we chose also includes its own limitation. Since we compared EQ-VAS and EQ-5D-3L scores after adjusting participants’ background by IPW method with propensity score, we could include most of the participants in the main analysis. Nevertheless, we had to exclude some of them due to missing items, and these missing values might have some impact on the result. Additionally, variables we collected from the survey was limited, then there might be other factors which we could not take into consideration in this study. These limitations will be future challenges to be addressed. Nevertheless, we can consider our results were robust to some extent because both ATE evaluation and linear regression analysis showed similar results. They both indicate that the symptoms caused by long-COVID might impair our quality of life.

## Conclusions

What we call “long-COVID” brings us substantial disease burden in addition to the burden attributed to the acute phase of COVID-19. This additional burden makes the whole disease burden of COVID-19 heavier, making prevention strategies all the more important. The influence of acute phase treatment, vaccination, and variants on “long-COVID” should be examined in the near future.

## Data Availability

The data that support the findings of this study are available upon request to the corresponding author. The data are not publicly available due to privacy or ethical restrictions.

## References

[1] Guan W, Ni Z, Hu Y, Liang W, Ou C, He J (2020). Clinical characteristics of coronavirus disease 2019 in China. N Engl J Med.

[2] Nozaki I, Miyano S (2020). The necessity of continuous international cooperation for establishing the coronavirus disease 2019 diagnostic capacity despite the challenges of fighting the outbreak in home countries. GHM.

[3] del Rio C, Collins LF, Malani P (2020). Long-term health consequences of COVID-19. JAMA.

[4] UK guidelines for managing long-term effects of COVID-19. Lancet. https://www.thelancet.com/journals/lancet/article/PIIS0140-6736(21)00847-3/fulltext 10.1016/S0140-6736(21)00847-3 PMC8102038 33965081

[5] Lopez-Leon S, Wegman-Ostrosky T, Perelman C, Sepulveda R, Rebolledo PA, Cuapio A (2021). More than 50 long-term effects of COVID-19: a systematic review and meta-analysis. Sci Rep.

[6] Sivan M, Taylor S (2020). NICE guideline on long Covid. BMJ.

[7] Crook H, Raza S, Nowell J, Young M, Edison P (2021). Long Covid-mechanisms, risk factors, and management. BMJ.

[8] Miyazato Y, Morioka S, Tsuzuki S, Akashi M, Osanai Y, Tanaka K (2020). Prolonged and late-onset symptoms of coronavirus disease 2019. Open Forum Infect Dis.

[9] Mandal S, Barnett J, Brill SE, Brown JS, Denneny EK, Hare SS (2021). ‘Long-COVID’: a cross-sectional study of persisting symptoms, biomarker and imaging abnormalities following hospitalisation for COVID-19. Thorax.

[10] Carfì A, Bernabei R, Landi F, Gemelli Against COVID-19 Post-Acute Care Study Group (2020). Persistent symptoms in patients after acute COVID-19. JAMA.

[11] Goërtz YMJ, Herck MV, Delbressine JM, Vaes AW, Meys R, Machado FVC (2020). Persistent symptoms 3 months after a SARS-CoV-2 infection: the post-COVID-19 syndrome?. ERJ Open Res.

[12] Tenforde MW (2020). Symptom duration and risk factors for delayed return to usual health among outpatients with COVID-19 in a multistate health care systems network—United States, March–June 2020. MMWR Morb Mortal Wkly Rep.

[13] Mazza MG, De Lorenzo R, Conte C, Poletti S, Vai B, Bollettini I (2020). Anxiety and depression in COVID-19 survivors: role of inflammatory and clinical predictors. Brain Behav Immun.

[14] Halpin SJ, McIvor C, Whyatt G, Adams A, Harvey O, McLean L (2021). Postdischarge symptoms and rehabilitation needs in survivors of COVID-19 infection: a cross-sectional evaluation. J Med Virol.

[15] Boscolo-Rizzo P, Borsetto D, Fabbris C, Spinato G, Frezza D, Menegaldo A (2020). Evolution of altered sense of smell or taste in patients with mildly symptomatic COVID-19. JAMA Otolaryngol Head Neck Surg.

[16] Puntmann VO, Carerj ML, Wieters I, Fahim M, Arendt C, Hoffmann J (2020). Outcomes of cardiovascular magnetic resonance imaging in patients recently recovered from coronavirus disease 2019 (COVID-19). JAMA Cardiol.

[17] Malik P, Patel K, Pinto C, Jaiswal R, Tirupathi R, Pillai S (2022). Post-acute COVID-19 syndrome (PCS) and health-related quality of life (HRQoL)—a systematic review and meta-analysis. J Med Virol US.

[18] Tran V-T, Riveros C, Clepier B, Desvarieux M, Collet C, Yordanov Y (2021). Development and validation of the long Covid symptom and impact tools, a set of patient-reported instruments constructed from patients’ lived experience. Clin Infect Dis.

[19] Tabacof L, Tosto-Mancuso J, Wood J, Cortes M, Kontorovich A, McCarthy D (2022). Post-acute COVID-19 syndrome negatively impacts physical function, cognitive function, health-related quality of life and participation. Am J Phys Med Rehabil.

[20] Fink TT, Marques HHS, Gualano B, Lindoso L, Bain V, Astley C (2021). Persistent symptoms and decreased health-related quality of life after symptomatic pediatric COVID-19: a prospective study in a Latin American tertiary hospital. Clinics (Sao Paulo).

[21] Matsunaga N, Hayakawa K, Terada M, Ohtsu H, Asai Y, Tsuzuki S (2020). Clinical epidemiology of hospitalized patients with COVID-19 in Japan: Report of the COVID-19 REGISTRY JAPAN. Clin Infect Dis.

[22] Cohen J (1988). Statistical power analysis for the behavioral sciences.

[23] R Core Team (2018). R: a language and environment for statistical computing.

[24] Rabin R, Gudex C, Selai C, Herdman M (2014). From translation to version management: a history and review of methods for the cultural adaptation of the EuroQol five-dimensional questionnaire. Value Health.

[25] Tsuchiya A, Ikeda S, Ikegami N, Nishimura S, Sakai I, Fukuda T (2002). Estimating an EQ-5D population value set: the case of Japan. Health Econ.

[26] Rosenbaum PR, Rubin DB (1983). The central role of the propensity score in observational studies for causal effects. Biometrika.

[27] Dolan P (1997). Modeling valuations for EuroQol health states. Med Care.

[28] Zhang Z, Kim HJ, Lonjon G, Zhu Y, Group written on behalf of AB-DCTC (2019). Balance diagnostics after propensity score matching. Ann Transl Med..

[29] Johnston R, Jones K, Manley D (2018). Confounding and collinearity in regression analysis: a cautionary tale and an alternative procedure, illustrated by studies of British voting behaviour. Qual Quant.

[30] Mcclure NS, Sayah FA, Xie F, Luo N, Johnson JA (2017). Instrument-defined estimates of the minimally important difference for EQ-5D-5L Index Scores. Value Health.

[31] Pastor-Barriuso R, Pérez-Gómez B, Hernán MA, Pérez-Olmeda M, Yotti R, Oteo-Iglesias J (2020). Infection fatality risk for SARS-CoV-2 in community dwelling population of Spain: nationwide seroepidemiological study. BMJ.

[32] Poletti P, Tirani M, Cereda D, Trentini F, Guzzetta G, Marziano V (2020). Age-specific SARS-CoV-2 infection fatality ratio and associated risk factors, Italy, February to April 2020. Eurosurveillance.

[33] van Asten L, Harmsen CN, Stoeldraijer L, Klinkenberg D, Teirlinck AC, de Lange MMA (2021). Excess deaths during influenza and coronavirus disease and infection-fatality rate for severe acute respiratory syndrome coronavirus 2, the Netherlands. Emerg Infect Dis.

[34] Tsuzuki S, Yoshihara K (2020). The characteristics of influenza-like illness management in Japan. BMC Public Health.

[35] Carson G, Carson G, Sigfrid L, Olliaro P, Norton A, Paparella G (2021). Research priorities for long Covid: refined through an international multi-stakeholder forum. BMC Med.

[36] Escobar LE, Molina-Cruz A, Barillas-Mury C (2020). BCG vaccine protection from severe coronavirus disease 2019 (COVID-19). PNAS Natl Acad Sci.

[37] Pilishvili T (2021). Interim estimates of vaccine effectiveness of Pfizer-BioNTech and Moderna COVID-19 vaccines among health care personnel—33 U.S sites, January–March 2021. MMWR Morb Mortal Wkly Rep.

[38] Polack FP, Thomas SJ, Kitchin N, Absalon J, Gurtman A, Lockhart S (2020). Safety and efficacy of the BNT162b2 mRNA Covid-19 vaccine. N Engl J Med.

[39] Modes ME, Directo MP, Melgar M, Johnson LR, Yang H, Chaudhary P (2022). Clinical characteristics and outcomes among adults hospitalized with laboratory-confirmed SARS-CoV-2 infection during periods of B.1.617.2 (Delta) and B.1.1.529 (Omicron) variant predominance — One Hospital, California, July 15–September 23, 2021, and Dec. MMWR Morb Mortal Wkly Rep.

[40] Bouzid D, Visseaux B, Kassasseya C, Daoud A, Fémy F, Hermand C, et al. Comparison of patients infected with delta versus omicron COVID-19 variants presenting to Paris Emergency Departments. Ann Intern Med. 2022. 10.7326/m22-0308. 10.7326/M22-0308 PMC8941485 35286147

